# Persistent Interruption in Parvalbumin-Positive Inhibitory Interneurons: Biophysical and Mathematical Mechanisms

**DOI:** 10.1523/ENEURO.0190-24.2024

**Published:** 2024-07-04

**Authors:** Carol M. Upchurch, Christopher J. Knowlton, Simon Chamberland, Carmen C. Canavier

**Affiliations:** ^1^Department of Cell Biology and Anatomy, Louisiana State University Health Sciences Center, New Orleans, Louisiana 70112; ^2^Neuroscience Institute and Department of Neuroscience and Physiology, New York University Langone Medical Center, New York 10016

**Keywords:** fast-spiking interneuron, hippocampus, K_V_1, persistent activity

## Abstract

Persistent activity in excitatory pyramidal cells (PYRs) is a putative mechanism for maintaining memory traces during working memory. We have recently demonstrated persistent interruption of firing in fast-spiking parvalbumin-expressing interneurons (PV-INs), a phenomenon that could serve as a substrate for persistent activity in PYRs through disinhibition lasting hundreds of milliseconds. Here, we find that hippocampal CA1 PV-INs exhibit type 2 excitability, like striatal and neocortical PV-INs. Modeling and mathematical analysis showed that the slowly inactivating potassium current K_V_1 contributes to type 2 excitability, enables the multiple firing regimes observed experimentally in PV-INs, and provides a mechanism for robust persistent interruption of firing. Using a fast/slow separation of times scales approach with the K_V_1 inactivation variable as a bifurcation parameter shows that the initial inhibitory stimulus stops repetitive firing by moving the membrane potential trajectory onto a coexisting stable fixed point corresponding to a nonspiking quiescent state. As K_V_1 inactivation decays, the trajectory follows the branch of stable fixed points until it crosses a subcritical Hopf bifurcation (HB) and then spirals out into repetitive firing. In a model describing entorhinal cortical PV-INs without K_V_1, interruption of firing could be achieved by taking advantage of the bistability inherent in type 2 excitability based on a subcritical HB, but the interruption was not robust to noise. Persistent interruption of firing is therefore broadly applicable to PV-INs in different brain regions but is only made robust to noise in the presence of a slow variable, K_V_1 inactivation.

## Significance Statement

Persistent activity in neuronal networks is thought to provide a substrate for multiple forms of memory. The architecture of neuronal networks across many brain regions involves a small number of locally projecting inhibitory neurons that control many excitatory pyramidal neurons that provide the output of the region. We propose that persistent silencing of fast-spiking parvalbumin-expressing inhibitory interneurons (PV-INs) can result in persistent activity of pyramidal neurons. We use a mathematical approach and computer simulations to show how a slowly changing state of a particular ion channel controls the long-lasting silence imposed by persistent interruption. Overall, our results provide a conceptual framework that positions the persistent interruption of PV-IN firing as a potential mechanism for persistent activity in pyramidal cells.

## Introduction

The hippocampus has been implicated in short-term memory tasks (Leavitt et al., 2017) in which information retention is necessary for a few hundreds of milliseconds (Weiss et al., 1999) to tens of seconds (Hampson and Deadwyler, 2000; Hampson et al., 2004) in the absence of sensory stimulation. For example, cue-associated activity persists during the delay period of delayed response tasks in multiple regions of the brain (Leavitt et al., 2017), including the hippocampus (Watanabe and Niki, 1985; Cahusac et al., 1989). A possible substrate for the representation of information in the absence of a sensory stimulus is persistent activity, defined as “a sustained change in action potential discharge that long outlasts a stimulus” (Major and Tank, 2004). Multiple mechanisms both at the network and at the single-cell level have been proposed to underlie persistent neuronal activity. One proposed mechanism for persistent activity is a mnemonic attractor sustained by reverberatory dynamics through feedback loops in a neural assembly (Wang, 2021). Another putative substrate for persistent activity relies on the intrinsic bistable dynamic of individual neurons (Zylberberg and Strowbridge, 2017). On the single-cell level, persistent Na^+^ current (Yamada-Hanff and Bean, 2013) in CA1 pyramidal cells (PYRs), Ca^2+^-activated Ca^2+^ currents in entorhinal cortical PYRs (Egorov et al., 2002) and CA1 PYRs (Combe et al., 2023), and L-type Ca^2+^ currents in other cells (Otsuka et al., 2001; Perrier et al., 2002) have been shown to contribute to regenerative firing, by making the neuron bistable or even multistable, and thus more likely to continue firing after excitation is terminated.

Neuronal discharge was originally described in terms of firing continuity as a function of continuous current injection. Neurons with Hodgkin's type 1 excitability can fire arbitrarily slowly and have a continuous frequency/current (*f*/*I*) relationship. In contrast, neurons with Hodgkin's type 2 excitability show repetitive firing of action potentials that cannot be sustained below a threshold firing frequency, and the *f*/*I* relationship is discontinuous at the value of applied current that is sufficient to sustain repetitive firing (Hodgkin, 1948). The determinant of the excitability type is whether inward or outward currents are dominant at equilibrium at the values of membrane potential traversed during the interspike interval (ISI). In type 1, inward currents dominate, whereas in type 2 outward currents dominate at equilibrium. Therefore, the interspike interval must be short enough that the slower outward currents do not come to equilibrium, resulting in a lower bound on the frequency that can be sustained during repetitive firing. Neurons with type 2 excitability display inherent bistability [either from a subcritical Hopf bifurcation (HB) or a saddle node but not on an invariant circle bifurcation (Izhikevich, 2007)] near the abrupt onset of tonic firing. If the region of bistability is large enough to be experimentally observable, it could theoretically contribute to switch-like transitions in neuronal networks. Here we examine whether and how different forms of bistability can sustain persistent activity.

We suggest a mechanism for persistent activity in PYRs involving the persistent silencing of presynaptic interneurons in a simple feedforward inhibitory circuit. Recent work has shown that a GABA_A_-mediated inhibitory postsynaptic potential can interrupt tonic firing in hippocampal parvalbumin-positive interneurons (PV-INs) for hundreds of milliseconds after the IPSP has dissipated (Chamberland et al., 2023). The interruption of firing is dependent upon K_V_1 because the interruption is blocked by the application of the specific Kv1 blockers dendrotoxin-K and I (Chamberland et al., 2023). The resulting long-lasting silence in hippocampal PV-INs tripled the firing rate of postsynaptic CA1-PYRs. Therefore, the mechanisms controlling firing interruption in hippocampal PV-INs could be key in understanding persistent activity in downstream CA1-PYRs.

Here, we used single-compartment, conductance-based computational models of PV-INs to describe the persistent interruption of firing dynamics. The model replicated type 2 excitability and explained the different firing regimes experimentally observed in CA1 hippocampal PV-INs. Bifurcation analysis and a fast/slow separation of time scales approach (Bertram and Rubin, 2017) revealed a subcritical HB dependent on the slow dynamics of K_V_1 inactivation. Although K_V_1 was not necessary for firing interruption in a medial entorhinal cortex (mEC) PV-IN model, it conferred significant robustness to noise.

## Materials and Methods

### Acute hippocampal slice preparation and electrophysiological recordings

All experiments performed were approved by NYU Langone IACUC protocols. Acute brain slices were prepared from P20–P35 male and female mice. *Pv*-Ai9 animals were obtained by crossing Pv-Cre mice (B6;129P2-Pvalb^tm1(cre)Arbr^/J, JAX stock #017320) to Ai9 reporter line mice (B6.Cg-Gt(ROSA)26Sor^tm9(CAG-tdTomato)Hze^/J, JAX stock #007909). Mice were deeply anesthetized with isoflurane and decapitated. The brain was extracted and placed in an ice-cold oxygenated (95% O_2_ and 5% CO_2_) sucrose ACSF solution. All compounds for electrophysiological solutions came from Sigma-Aldrich. The sucrose ACSF solution contained the following (in mM): sucrose 185 (CAS 57-50-1), NaHCO_3_ 25 (CAS 144-55-8), KCl 2.5 (CAS 7447-40-7), NaH_2_PO_4_ 1.25 (CAS 7558-80-7), MgCl_2_ 10 (CAS 7791-18-6), CaCl_2_ 0.5 (CAS 10035-04-8), and glucose 25 (CAS 50-99-7), pH = 7.4, 330 mOsm. The brain was dissected and transverse acute hippocampal slices (300 µm) were prepared on a vibratome (Leica VT1000 S). Hippocampal slices were transferred to an ACSF-containing recovery chamber that was continuously oxygenated and maintained at 32°C. The ACSF contained the following (in mM): NaCl 125 (CAS 7647-14-5), NaHCO_3_ 25, KCl 2.5, MgCl_2_ 2, CaCl_2_ 2, and glucose 10, pH = 7.4, 300 mOsm. After a recovery period of 30 min, the temperature was allowed to come down to room temperature for the rest of the experiments. Experiments were started after at least 1 h after slice transfer to the recovery chamber. For electrophysiological recordings, acute slices were transferred to a submerged recording chamber under an upright microscope (BX61WI, Olympus) equipped with a water-immersion 40× objective (Zeiss). TdTomato + interneurons located in the CA1 stratum oriens bordering the PYR layer were visually identified and selected for whole-cell recordings. Recording electrodes were prepared from borosilicate glass filaments (TW150-4, World Precision Instruments) using a P-97 Sutter Instrument micropipette puller. Recording electrodes were filled with a K+-based intracellular solution that contained the following (in mM): 130 K-gluconate (CAS 299-27-4), 10 HEPES (CAS 7365-45-9), 2 MgCl_2_, 2 Mg_2_ATP (CAS 74804-12-9), 0.3 NaGTP (CAS 36051-31-7), 7 Na_2_-phosphocreatine (CAS 19333-65-4), 0.6 EGTA (CAS 67-42-5), and 5 KCl. The pH was adjusted to 7.2 using 1 M KOH, and the final solution had an osmolality of 295 mOsm. Recording electrodes had a resistance of 3–6 MΩ. The electrophysiological signal was amplified with an Axopatch 200B and digitized at 10 kHz with a Digidata 1322A (Axon Instruments). Current-clamp recordings consisted of a 500 ms depolarizing current step of increasing amplitude, delivered over multiple trials every 10 s. The data was recorded on a personal computer. The liquid junction potential (estimated at ∼15 mV) was not corrected. Hippocampal slice recordings were conducted at room temperature, and the model was calibrated using this data.

### Analysis of electrophysiological data

The input resistance was measured using a voltage step from −60 to −50 mV. The time constant was found using a current step yielding an ∼10 mV change in membrane potential and assuming a monoexponential fit. The resulting average capacitance was calculated using the input resistance and the time constant: τ = RC. The minimum firing frequencies for type 2 excitability were determined using the average frequency of episodes of regular spiking, whether sustained or stuttering, at the lowest level of current that supported these episodes. Traces that had interspike intervals that were >1.5 times the average interspike interval were inspected for stuttering. If the trace showed stuttering, the long ISIs were removed from the average.

### Computational model

We began with the PV-IN model from Chamberland et al. (2023). The differential equation for the membrane potential (V in mV) for the neuron is given by the following:

$$C_{M}dV/dt=\;-I_{\mathrm{App}}-I_{\mathrm{leak}}-I_{Kv3}-I_{Kv1}-I_{\mathrm{Na}},$$

where *C_M_* is the capacitance of the cell membrane (1 µF/cm^2^), *I*_App_ is the current injected, *I*_leak_ is the passive leak current, *I_Kv_*_3_ is the fast delayed rectifier, *I_Kv_*_1_ is the slowly inactivating current mediated by *K_V_*1, and *I*_Na_ is the fast sodium current. *I*_leak_ was described by the following equation:

$$I_{\mathrm{leak}}(v)=Ig_{L}*(v-E_{L}).$$

The differential equations for the gating variables are of the following form:

$$dx/dt=(x-x_{\inf}(v))//\tau_{x}(v).$$

*I_Kv_*_3_ was described by the following equations:

$$I_{Kv3}(v,n)=g_{Kv3}*n^{2}*(v-E_{K}),$$

where *n* relaxes to its instantaneous voltage state:

$$n_{\inf}(v)=\{1+\exp[-(v+12.4)/6.8]{\}}^{-1},$$

with time constant:

$$\tau_{n}(v)=\{0.087+11.4*{\left\{1+\exp[(v+14.6)/8.6]\right\}}^{-1}\}\;*\{0.087+11.4*{\left\{1+\exp[-(v-1.3)/18.7]\right\}}^{-1}\}\;,$$

and *g_Kv_*_3_ retained its original value of 0.223 mS/cm^2^. *Kv*3 channels are critical to models of fast-spiking interneurons because they deactivate very quickly enabling very fast spiking (Rudy et al., 1999; Rudy and McBain, 2001) and are present in fast-spiking PV-INs at a much higher level than in other cell types (Erisir et al., 1999). Their contribution to the afterhyperpolarization (AHP) also limits the accumulation of Na^+^ channel inactivation (Erisir et al., 1999).

The sodium current was described by the following:

$$I_{\mathrm{Na}}(v,m,h)=g_{\mathrm{Na}}*m^{3}*h*(V-E_{\mathrm{Na}}),$$

where *E*_Na _= 50 mV,

$$m_{\inf}(v)=\{1+\exp[-(v-V_{h})/11.5]{\}}^{-1},$$

τ*_m _*= 0.001 ms, $h_{\inf}(v)=\{1+\exp[(v+58.3)/6.7]{\}}^{-1}$,

$$\tau_{h}(v)=7*\{1+\exp[(v+60)/12]{\}}^{-1},$$

and *g*_Na_ retained its original value of 0.1125 mS/cm^2^. Table 1 gives parameters that were changed from the Chamberland model to more closely replicate the electrical activity of PV-INs in CA1. *I*_Kv1_ was described by the equation $I_{Kv1}=g_{Kv1}*p*q*(V-E_{K})$ where *E_K_*_ _= −90 mV. The steady state for the activation gate *p* was $p_{\inf}(v)=\{1+\exp[-(v+41.4)/26.6]{\}}^{-4}$ with time constant $\tau_{p}=0.448\;\mathrm{ms}$. The steady state for inactivation gate *q* was $q_{\inf}(v)=\{1+\exp[(v+78.5)/6]{\}}^{-1}$, and the time constant was $\tau_{q}(v)=k*(v+105)$. Simulations were run in Neuron (Hines and Carnevale, 1997), and the code is available at (https://modeldb.science/2016658).

**Table 1.** Parameters that were changed from Chamberland et al. (2023)

	Chamberland model	Modification
Length of cell	10 µm	126 µm
*g_L_*	0.1 mS/cm^2^	0.25 mS/cm^2^
*g_Kv_*_1_	10 mS/cm^2^	5 mS/cm^2^
*V_h_*	−24 mV	−22 mV
*K*	10	7.5

The model for the mEC interneuron was taken from the homogeneous network in Via et al. (2022) without modification.

### Bifurcation analyses

Bifurcation analyses were performed by using the MATCONT package (Dhooge et al., 2003).

## Results

### Model calibration

**Figure 1.**
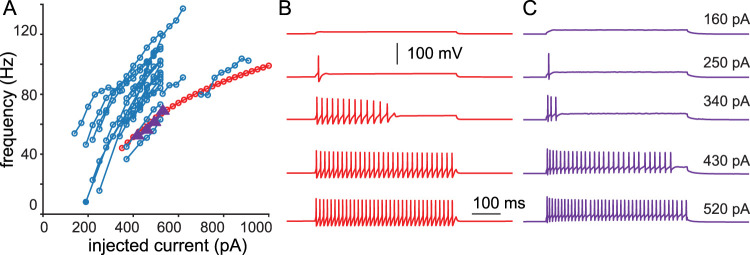
Frequency/current (*f*/*I*) relationship*. **A***, Experimental data points (blue circles, purple triangles). Model *f*/*I* curve (red circles). ***B***, Voltage traces in model neuron in response to step currents. ***C***, Voltage traces from experimental data points from a representative PV-IN marked by purple triangles in ***A***. At values of depolarizing current too low to support repetitive spiking, both model and real neurons emit one or more spikes and then fall silent.

To better capture the passive properties and the *f*/*I* relationship of hippocampal CA1 PV-INs, the model in Chamberland et al. (2023) was modified as described in the Materials and Methods. The resulting input resistance of the model was 80.6 MΩ, similar to that measured experimentally in vitro, 78.7 MΩ (SD = 24.4; *n* = 27). The model had a membrane time constant of 4.1 ms and a capacitance of 50.9 pF, which were at the low end of the experimental values (10.4 ± 4.9 ms, *n* = 27 and 142 pF ± 82.3, *n* = 27) but still physiologically plausible. Depolarizing steps of increasing amplitude revealed a nonlinear relationship between firing frequency and current injection in the model, closely aligned with experimental observations (Fig. 1*A–C*). The progression from nonspiking or transient firing to sustained high-frequency firing resulting in discontinuous *f*/*I* curves in PV-INs is consistent with type 2 excitability as originally described by Hodgkin (Hodgkin, 1948), a feature which our model is the first to capture in CA1 PV-INs.

**Figure 2.**
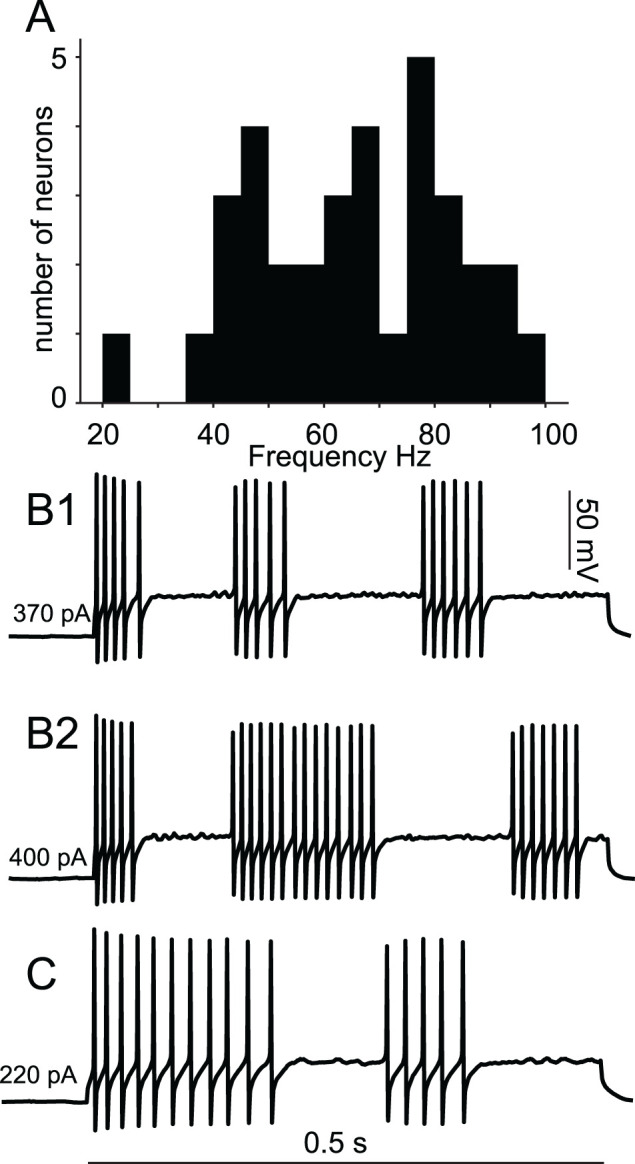
Evidence for Hodgkin's type 2 excitability. ***A***, Histogram of the minimum sustained firing frequency for PV-INs. ***B***, Two traces from the same neuron exhibiting stuttering and subthreshold oscillations. ***C***, As in ***B***, single trace from a neuron with lower cutoff frequency.

We examined the voltage traces used to determine the *f*/*I* curves in Figure 1*A*. Thirty-four of 35 PV-INs exhibited a frequency threshold below which they could not fire (Fig. 2*A*), which is the definition of Hodgkin's type 2 excitability. The neuron with the cutoff frequency at 20 Hz was the only instance that was ambiguous and might be consistent with type 1 excitability. At near-threshold current levels, long intervals of arbitrary duration were occasionally observed (Fig. 2*B,C*). After an initial brief ramp, the membrane potential during the long intervals was essentially flat, suggesting that the trajectory was governed by the stable fixed point that is inside the spiking trajectory near a subcritical HB. During most of these essentially flat intervals, a subthreshold oscillation can be observed, a hallmark of a HB. Whereas a subcritical HB is not necessary for type 2 excitability (Izhikevich, 2007), it is sufficient.

### Bifurcation analysis of persistent interruption

PV-IN firing can be persistently interrupted by brief membrane hyperpolarization, leaving the neuron in a depolarized quiescent state. We next aimed to obtain a mathematical understanding of the transitions between firing and silent states in PV-INs. The mechanisms underlying transitions between spiking and quiescence can be understood using a bifurcation diagram that graphs the transition points in a space consisting of a fast variable on the *y*-axis and a slow variable on the *x*-axis (Izhikevich, 2007).

**Figure 3.**
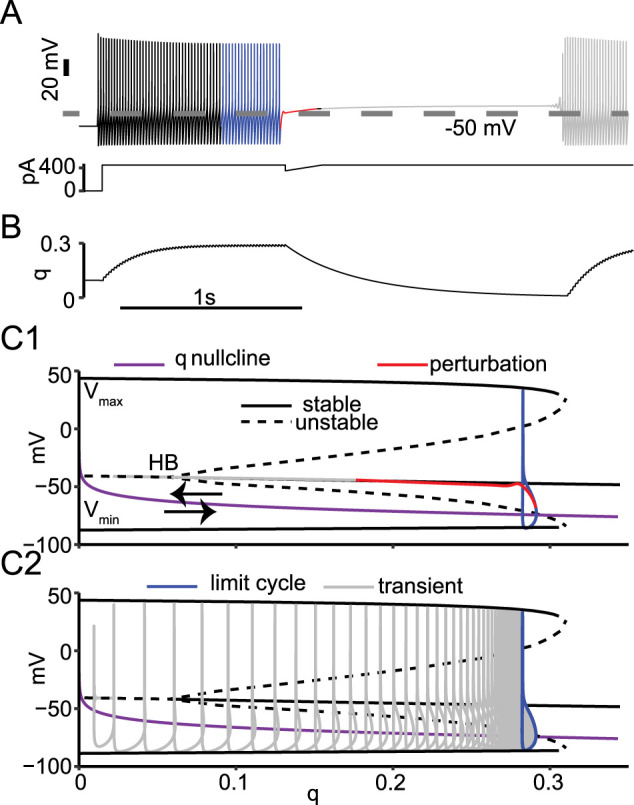
Bifurcation analysis of persistent interruption. ***A***, Persistent interruption of tonic firing by a mock IPSP in a model PV-IN. ***B***, Time course of K_V_1 inactivation gate. ***C***, Bifurcation diagram with *q* as the bifurcation parameter. ***C*1**, Leftward movement of trajectory during perturbation and interruption. ***C*2**, Rightward movement of trajectory during transient tonic spiking. See Extended Data Figures 3.1 and 3.2 for more details.

Our model replicated the interruption (Fig. 3*A*, top) using a simulated current-clamp waveform (Fig. 3*A*, bottom). The model neuron is initially silent, but a 450 pA current step induces tonic firing (Fig. 1*B*) that eventually reaches a steady state (Fig. 3*A*, blue). During tonic firing, the available fraction of the Kv1 channel (*q*) gradually accumulates until it reaches the steady state where the spike AHP removes the exact amount of inactivation that occurs during the depolarized part of the action potential. At this point, the value of *q* in Figure 3*B* levels out, with a small residual oscillation around a steady value. Extended Data Figure 3.1 also plots the activation variable *p* and the product pq; the inactivation variable *q* clearly controls the dynamics. The applied current was reduced sharply to 350 pA and linearly increased back to 450 pA during a 200 ms ramp to simulate an IPSP (a mock IPSP; Chamberland et al., 2023). The neuron was silenced during the mock IPSP (Fig. 3*A*, red trajectory) but also remained silent for hundreds of milliseconds after the end of the mock IPSP (gray curve). Subthreshold oscillations preceded the resumption in firing in experiments and in the model (see Fig. 5*A,D* and Supplementary Fig. 7E in Chamberland et al., 2023), suggesting the emergence of a subcritical HB (Izhikevich, 2007). The simulations in Figure 3 were run at room temperature (24°C) for parallelism with the experiments, but the persistent interruptions were robust at higher temperatures (Extended Data Fig. 3.2), consistent with experiments performed at higher temperatures (Fig. S1*G–I* in Chamberland et al., 2023).

10.1523/ENEURO.0190-24.2024.f3.1 Figure 3.1 Fig. 3B repeated with panels added for p and pq. Download Figure 3.1, TIF file.

10.1523/ENEURO.0190-24.2024.f3.2 Figure 3.2 Fig 3AB rerun at 32^o^ from a baseline of 23°C with Q10 of 2. Persistent interruption is robust at higher temperatures, consistent with Chamberland et al 2023. The major effect of increasing the temperature was to shift the Hopf bifurcation to more hyperpolarized potentials. Download Figure 3.2, TIF file.

To better understand the firing interruption and firing resumption transitions in the system, we performed a bifurcation analysis (Fig. 3*C*) using fast/slow time scale analysis (Bertram and Rubin, 2017). Since the inactivation gate for K_V_1 (*q*) builds up slowly during tonic firing and decays slowly during the firing interruption, we used *q* as a parameter in the bifurcation analysis. The bifurcation analysis holds the slow parameter constant to find the points at which transitions occur. The output of the bifurcation analysis was a set of fixed points (resting membrane potentials), which could be stable (solid black curves) or unstable (dashed black curves), and the maxima and minima of oscillations in membrane potential (limit cycles). The subcritical HB (Fig. 3*C*1) is at the transition point between the branches of fixed points. The *q* nullcline (Fig. 3*C*, purple curve), which is the steady value of *q* at each value of the membrane potential, separates the regions of state space where *q* is increasing (below) and decreasing (above). These results show the dual role of *q* in establishing a stable firing state and an unstable dynamic state at the subcritical HB.

Next, we consider *q* as a dynamic variable to understand how the bifurcation structure drives the model trajectory. We superimposed the trajectory of the model from Figure 3, *A* and *B*, into the *q*/*V* plane in Figure 2, *C*1 and *C*2, discarding the initial firing (Fig. 3*A*, black trace) and beginning at the steady-state oscillation shown in blue in Figure 3, *A* and *C*. A major modification in the Chamberland et al. (2023) model compared with the Golomb et al. (2007) model is in the description of K_V_1. In Golomb et al. (2007), the inactivation time constant (τ*_q_*) for K_V_1 is 150 ms at all membrane potentials. In Chamberland et al. (2023) and in this study, the equation for τ*_q_* depends linearly on the membrane potential, so it is large (slow) at relatively depolarized potentials (>−55 mV), but faster during the AHP at hyperpolarized potentials. The *q* nullcline divides the *q*/*V* plane into two halves. Below the nullcline, in the simulations, *q* increases rapidly, and the trajectory moves to the right. Above the *q* nullcline, *q* slowly decreases, moving the trajectory to the left. This dynamic picture allows us to make sense of the model activity, as explained below.

At the subcritical HB (Izhikevich, 2007), the fixed point (resting membrane potentials) transitions from unstable (dashed line) to stable (solid curve obscured by the red and gray trajectory curves in Fig. 3*C*1 but visible in Fig. 3*C*2). In the bifurcation diagram, an oscillation is described only by the maximum and minimum values of the membrane potential achieved. An oscillation that traces an identical cycle on each oscillation is called a limit cycle. An unstable limit cycle (indicated by the two dashed curves representing the maxima and minima) which is not robust to noise (Izhikevich, 2007) emerges from the subcritical HB. The unstable limit cycle becomes a stable oscillation representing tonic firing (maxima and minima indicted by the two solid curves) and forms the boundary between the stable repetitive firing and a stable resting membrane potential that coexist at values of *q* held constant between ∼0.07 and 0.3. This coexistence is called bistability. A neuron that starts its trajectory outside of this unstable limit cycle will continue firing on the stable limit cycle, but trajectories within the unstable limit cycle move toward the fixed point. Since the model is bistable, the interruption (shown in red) pulls the model's trajectory within the unstable limit cycle and lands on the branch of stable fixed points representing the depolarized quiescent state. In repetitive spiking, the fast Na^+^ current produces an action potential upstroke before the K^+^ currents can activate sufficiently (and the Na^+^ channel inactivates sufficiently) to prevent the action potential. Although the neuron is depolarized beyond the action potential threshold observed during repetitive firing, it remains silent because the slow return to the baseline value of injected current allowed the fast variables to reach a steady state in which the outward currents dominate. The trajectory then moves slowly to the left through a series of stable fixed points (that are only stable at a constant value of *q*) as *q* decays. This series of “stable” fixed points was referred to by Chamberland et al. (2023) as a “drifting stable point in the membrane potential”; here we show that the drift is caused by the slow dynamic evolution of the *q* variable, resulting in an interruption that outlasts the mock IPSP. After *q* decays below the HB, the trajectory travels along the weakly repelling branch of unstable fixed points, which prolongs the interruption, until the repulsion becomes obvious as an oscillation that starts with small amplitude (Fig. 3*C*1, inset) and grows. Figure 3*C*2 illustrates the trajectory once action potential firing commences, recapitulating the transient activity (gray trace) as *q* increases moving the trajectory to the right, until steady repetitive firing (limit cycle shown in blue) is achieved.

### Elliptical bursting

Elliptical bursting (Wang and Rinzel, 1995; Rinzel and Ermentrout, 1998), originally called type 3 bursting (Rinzel, 1987; Bertram et al., 1995), requires a slow variable to drive the intrinsic dynamics back and forth across a subcritical HB. PV-INs can undergo firing states reminiscent of elliptical bursting, sometimes referred to as “stuttering” (Bracci et al., 2003; Markram et al., 2004; Sciamanna and Wilson, 2011; Chamberland et al., 2023). We next explored if transitions between firing and silent states occur similarly as to the firing interruption.

**Figure 4.**
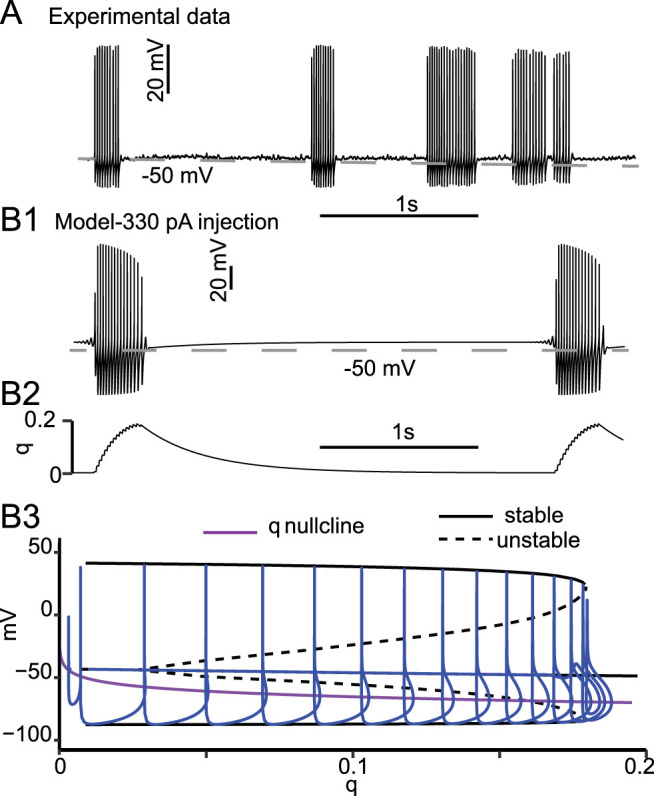
Elliptical bursting. ***A***, Experimentally observed trace recorded in bicuculline (10 μM) and CGP-55845 (2 μM). Experimentally observed elliptic bursting is less regular than that of a noiseless model because of the physiologically unavoidable noise, although these experiments were performed in the presence of blockers of inhibitory synaptic transmission. ***B*1**, Model membrane potential showing repetitive elliptical bursting with the same parameters as Figures 1*C* and 2 except that the injected current was 330 pA. ***B*2**, Time course of *q*. ***B*3**, Model trajectory for a single burst superimposed on the bifurcation diagram.

The model exhibited elliptical bursting in response to moderate current injection (Fig. 4*B*1), consistent with the observation of noisy elliptic putative bursting in experiments (Fig. 4*A*). The availability of K_V_1 indicated by the gating variable *q* (Fig. 4*B*2) controls the timing of the bursts. Figure 4*B*2 shows the bifurcation diagram with *q* held constant at an injected current of 330 pA. As in Figure 3*C* (calculated at 450 pA injected current), the inactivation gating variable *q* accumulates rapidly during the AHP that drops below that nullcline and then decays slowly during the subsequent depolarization and action potential. However, unlike in Figure 3*C*, a tonic, steady firing rate is never achieved. Instead, during spiking *q* continues to increase until the branch of stable limit cycles (solid curves representing the maxima and minima of the membrane potential) collides with the branch of unstable limit cycles at a bifurcation called a saddle node of periodics (Izhikevich, 2000). Since no stable limit cycle exists above a *q* value of ∼0.18 (for this level of current injection), the trajectory collapses onto the branch of stable fixed points (not visible under the blue trajectory but similar to that seen in Fig. 3*C*2). The trajectory then slowly relaxes to the left as the fixed point is above the *q* nullcline along a trajectory of stable fixed points (with respect to a fixed *q*) as *q* decays. When *q* decays past the HB, the stable membrane resting potential becomes unstable, initiating another cycle of spiking (Knowlton et al., 2020). These results show that PV-INs are silenced by distinct mechanisms during the firing interruption and elliptical bursting, while the return to the firing state is similarly explained in both cases by a drift away from the HB driven by the slow dynamic evolution of *q*.

### Parameter space compared with the neocortical PV-IN model

The above results implicate the existence of different firing regimes in CA1 PV-INs, as previously described in a model of neocortical PV-INs (Golomb et al, 2007). Given that this model was based on Golomb et al. (2007), we next explored the model to obtain a description of the different firing regimes in CA1 PV-INs.

**Figure 5.**
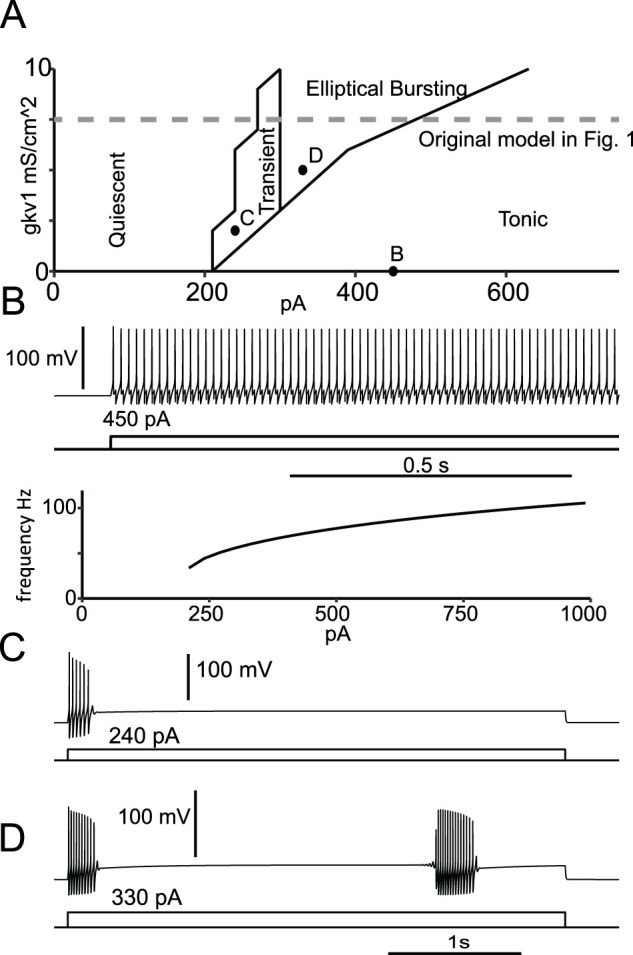
Parameter space. ***A***, Phase diagram in the plane of *g*_KV1_ and *I*_app_. ***B***, Top: tonic firing at 0 mS/cm^2^ and 450 pA Bottom: discontinuous *f*/*I* curve at 0 mS/cm^2^ characteristic of type 2 excitability (see text). ***C***, Transient firing at 2 mS/cm^2^ and 240 pA. ***D***, Elliptic bursting at 5 mS/cm^2^ and 330 pA repeated from Figure 4*B*1.

We first explored the parameter space in the plane of *g*_KV1_ and *I*_app_. We varied the injected current from 0 to 1,000 pA in 30 pA increments, and the conductance of K_v_1 from 0 to 10 mS/cm^2^ in increments of 1 mS/cm^2^. Similar to Figure 2 of Golomb et al. (2007), we observed four different firing regimes (Fig. 5*A*): either the model was quiescent, fired transiently, exhibited elliptical bursting, or fired continuously. Figure 5*B* shows an *f*/*I* curve with *g*_KV1_ set to zero (bottom) and an example of tonic firing (top). Since the *f*/*I* curve is discontinuous with an abrupt onset of firing at ∼40 Hz, in our model, K_V_1 is not required for type 2 excitability. Figure 5*C* gives an example of a transient response. In contrast to Golomb et al. (2007), transient spiking prior to quiescence was always observed instead of a delay prior to initiation of tonic spiking. This difference is explained by the description of K_V_1. Since there is some overlap in the steady-state activation and inactivation curves for K_V_1, there is a small steady-state “window” current at membrane potentials near the resting membrane potential. Here, K_V_1 has a much smaller window current and therefore a much smaller fraction of available K_V_1 at rest. In the Golomb et al. (2007) model, there is sufficient K_V_1 available at rest to silence the cell for sufficiently small values of *I*_app_, hence explaining the delay. Firing can only commence after the inactivation of the K_V_1 channel at the more depolarized potential resulting from the injected current. With less K_V_1 available at rest, there is insufficient K_V_1 current to stop the model from firing with the initial current injection. During the initial bout of spiking, the inactivation is removed during the AHP, allowing outward K_V_1 current to accumulate and render the model quiescent. This finding reconciles observations that PV-INs can exhibit delayed firing or fire at the onset of the depolarizing pulse. Considering that K_V_1 properties can be activity dependent, this would provide a likely explanation for differences between PV-INs. Figure 5*D* gives an example of elliptic bursting. Elliptical bursting in both models required a minimum amount of *g*_Kv1_. In contrast to Golomb et al., (2007), elliptic bursting began in the spiking phase rather than the quiescent phase. The difference in window current at rest also accounts for the lack of delay prior to initiating spiking during elliptic bursting in our model. Despite this difference, our simulations showed that the Golomb model in Figure 2*C* from that paper could also exhibit persistent interruption (data not shown).

### HB parameter: applied current versus slow dynamic variable

To understand how widespread the interruption of firing is across PV-IN models, we challenged a previously published model from the mEC with similar simulation paradigms (Via et al., 2022). Both models exhibit type 2 excitability, identified by the subcritical HB with *I*_app_ as the bifurcation parameter; this bifurcation occurs at the value of *I*_app_ at which tonic spiking is initiated as *I*_app_ is increased. This bifurcation is unrelated to the subcritical HB in our model of a CA1 PV-IN that occurs considering *q* as the bifurcation parameter. The subcritical HB that underlies type 2 excitability implies a region of bistability between spiking and quiescence at values of *I*_app_ on the stable side of the bifurcation.

**Figure 6.**
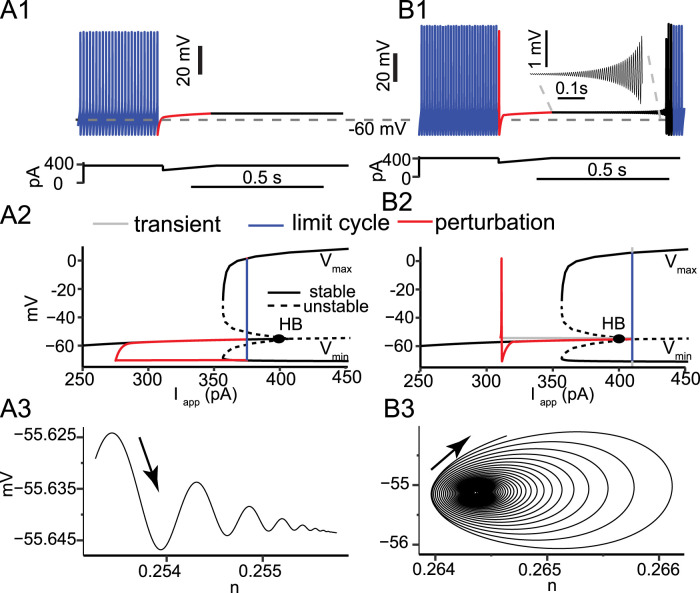
Perturbation from tonic firing to a stable versus an unstable branch of fixed points. The Via model at two levels of current, within the bistable regime (***A***) and a globally attracting tonic firing regime (***B***). ***A*1**, A mock IPSP of maximum amplitude of 100 pA that decayed linearly within 200 ms was subtracted from a constant applied current of 375 pA. ***A*2**, The trajectory was superimposed on the bifurcation structure of the model with applied current as the slow parameter. ***A*3**, Damped oscillatory trajectory in the phase plane of the *n* gate for K_V_3 and the voltage as the stable fixed point is approached during the transient following the interruption. ***B*1**, A similar mock IPSP was applied at a holding current of 410 pA. ***B*2**, Trajectory superimposed on the bifurcation structure. ***B*3**, Trajectory in the phase plane of the *n* gate for K_V_3 and voltage spiraling out from the branch of weakly repelling fixed points during the transient following the interruption.

At certain values of *I*_app_ and with an appropriately calibrated mock IPSP, a persistent interruption of firing can also be demonstrated in the (Via et al. 2022) model (Fig. 6*A*1,*B*1). Annihilation of firing that lasts beyond stimulus termination was first demonstrated in squid axon (Guttman et al., 1980), the very preparation in which Hodgkin and Huxley first modeled the generation of action potentials. Their classic model (Hodgkin and Huxley, 1952) exhibited type 2 excitability due to a subcritical HB, and this bistability was invoked to explain the observed cessation of firing in response to an appropriate perturbation. Therefore, both models of PV-INs could demonstrate the interruption of firing.

We next investigated the mechanisms controlling the interruption of firing in both models. In the EC PV-IN model at a holding current of 375 pA, a mock IPSP produces a permanent transition from tonic firing to quiescence in a noiseless model (Fig. 6*A*1). The bifurcation diagram in Figure 6*A*2 has *I*_app_, the applied current, as the bifurcation parameter instead of the gating variable *q* as in Figure 3*C* and 4*B*3. Figure 6*A*2 shows that at a holding current of 375 pA, the limit cycle representing tonic firing is just to the left of the subcritical HB; therefore, tonic firing is bistable with a stable resting membrane potential. The perturbation by the mock IPSP (red) moves the trajectory onto the stable branch of fixed points. Here, since *I*_app_ is a parameter that is held constant except during the perturbation, the fixed points are truly stable. Thus, in the absence of noise, the trajectory remains on the stable branch after the perturbation, with just a brief small amplitude transient (Fig. 6*A*3). In contrast, at an injected current of 410 pA, a mock IPSP elicits a persistent but finite interruption of firing (Fig. 6*B*1). The finite duration interruption is a consequence of the increased injected current shifting the limit cycle trajectory to the right of the HB (Fig. 6*B*2). Thus, the model is no longer bistable. The hyperpolarizing input (red) again moved the trajectory onto the branch of stable fixed points. However, once the trajectory crosses the HB as the applied current returns to its original depolarized value, the trajectory is forced to spiral out (Fig. 6*B*3) into the limit cycle that represents tonic repetitive firing. Although the branch of fixed points is unstable beyond the HB, it is only weakly repelling due to the small amplitude of the positive real parts of the eigenvalues at the fixed points (Izhikevich, 2007). The spiral is very slowly expanding (Fig. 6*B*1, inset), resulting in the persistent interruption.

**Figure 7.**
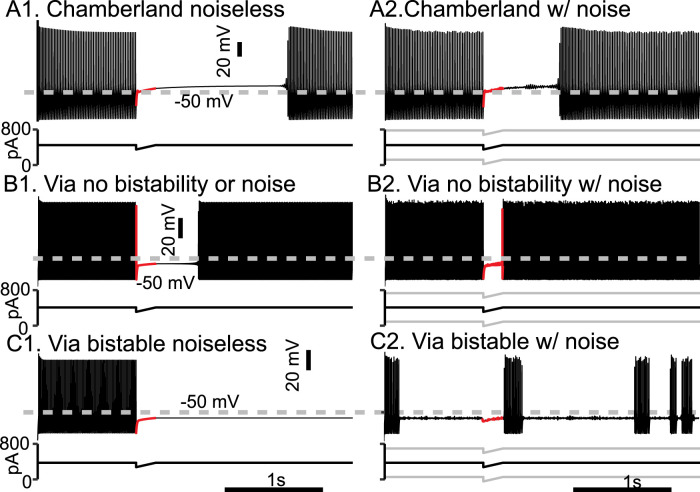
Robustness of interruptions to noise. The Chamberland model was compared with the Via model at two levels of current. The mock IPSP was 100 pA over 200 ms in all cases. The voltage waveform during the mock IPSP is shown in red. ***A*1**, Persistent interruption of the noiseless Chamberland model repeated from Figure 3*A*. ***A*2**, Same as A1 but added Gaussian current noise with a mean of 0 and a standard deviation of 164.7 pA. ***B*1**, Persistent interruption in the noiseless Via model repeated from Figure 6*B*1. ***B*2**, Same as ***B*1** but with additive Gaussian current noise with a standard deviation of 160.6 pA. ***C*1,** Persistent interruption in the Via model repeated from Figure 6*A*. ***C*2**, Same as ***B*1** but with additive Gaussian current noise with a standard deviation of 160.6 pA. Switches between firing and quiescence occur independently of the mock IPSP.

Our results suggest that the main effect of these differences would result in increased resilience to noise. To inject a comparable amount of noise in each model, we set the standard deviation of zero mean injected Gaussian current noise to the amount of current required to hyperpolarize each model by 5 mV (Destexhe and Rudolph-Lilith, 2012) from its HB point, assuming that *q* is held at its equilibrium value at the HB (0.05764) in the Chamberland model. This noise was added to the applied current waveform to compare the sensitivity of the different types of interruption to noise. In each case, 10 different noisy simulations were run. Figure 7*A*2 shows that the duration of the interruption in the Chamberland model from Figure 3*A* is still several hundreds of milliseconds (mean interruption length of 555.2 ms with standard deviation of 39.5 ms) in the presence of substantial current noise, although only about half the duration in a noiseless simulation (Fig. 7*A*1). In contrast, in the Via model in Figure 7*B*1 (repeated from Fig. 6*B*), which was not biased in the bistable range, the interruption vanishes (3.4 ms with a standard deviation of 16.9 ms) in the presence of substantial current noise (Fig. 7*B*2). The trajectory during the mock IPSP is shown in red, and in this example, firing resumes slightly before the mock IPSP terminates. In the Via model in Figure 7*C*1 (repeated from Fig. 6*A*), which was biased in the bistable regime, the interruption was also not robust to noise, but in a different way. In all 10 noisy simulations, including the one shown in Figure 7*C*2, an interruption was triggered by noise before the mock IPSP was delivered, and in 8 of 10 simulations, there were multiple transitions between spiking and quiescence. Therefore, we found that the mechanisms controlling the interruption of firing were not the same in the hippocampal and EC PV-IN models.

**Figure 8.**
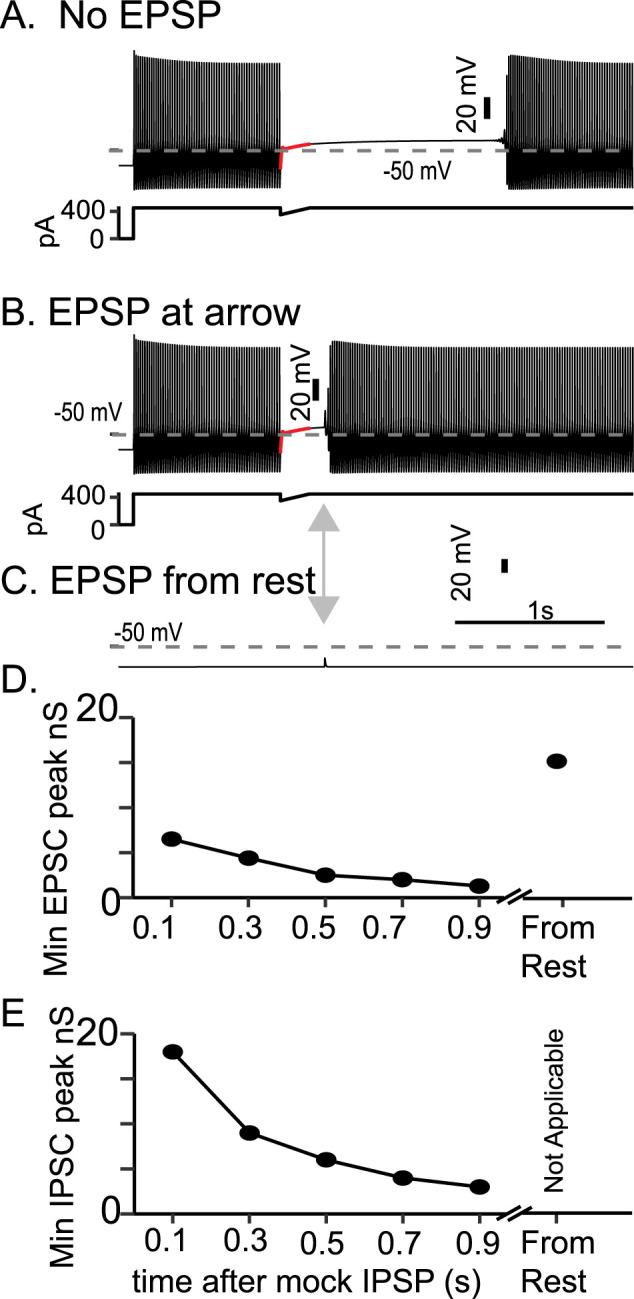
The quiescent state of the Chamberland model becomes more susceptible to synaptic input over time. The Chamberland model, when interrupted from rapid firing by an inhibitory mock IPSP, enters a quiescent state (***A***). Excitatory synaptic input given shortly after the end of the mock IPSP can return the model to firing (***B***). In ***B***, a biexponential conductance with a rise time of 0.5 ms, a fall time of 1 ms, a reversal potential of 0 mV, and a maximum conductance of 6.4 nS was enough to cause the model to return to firing. The same synapse given to the model at rest (***C***) did not. The minimum required conductance for the model to return to fire decreases during the interruption (***D***) and is higher at rest. Due to bistability with respect to the slow variable *q*, an inhibitory synapse with a reversal potential of −65 mV can also cause the model to fire from the quiescent state. The minimum required conductance for an inhibitory synapse also decreases with the duration of the interruption (***E***).

The level of noise or synaptic input required to cause the Chamberland model to return to firing is proportional to the size of the unstable limit cycle. The bifurcation diagrams in Figures 2*C* and 4*B*3 show that in the bistable regions, the unstable limit cycle forms the boundary between quiescence and tonic firing. In Figure 8*A* (repeated from Fig. 7*A*1 with the same parameter settings as Fig. 3*A*), the mock IPSP pulls the trajectory within the unstable limit cycle, and the model becomes quiescent. During this interruption, an additional excitatory postsynaptic conductance (EPSC) can push the trajectory back outside the unstable limit cycle, inducing firing as demonstrated in Figure 8*B*. The same EPSC is unable to induce firing in the model at rest (Fig. 8*C*). As *q* recovers, the unstable limit cycle shrinks, and the minimum peak conductance EPSC to induce firing decreases (Fig. 8*D*). Because the model is bistable, the required stimulus to induce firing is generally less than required at rest. The unstable limit cycle is a curve that surrounds the fixed points of the model both in the depolarizing and hyperpolarizing directions. Therefore, an inhibitory stimulus that pushes the trajectory past the hyperpolarized boundary of the unstable limit cycle will also induce firing. The required minimum conductance of this stimulus decreases during the interruption (Fig. 8*E*), due to the shrinking of the unstable limit cycle as *q* recovers.

## Discussion

### Summary

Persistent interruption of firing in PV-INs has been recently demonstrated (Chamberland et al., 2023). In this study, we used a fast/slow separation of times scales approach with the K_V_1 inactivation variable as the bifurcation parameter to show that an inhibitory postsynaptic potential can stop repetitive firing by moving the trajectory onto a coexisting stable fixed point corresponding to a depolarized quiescent state. Although the interruption could be observed in all models of PV-INs tested, the slow inactivation of K_V_1 at depolarized potentials made persistent interruptions resistant to noise and enabled elliptical bursting at some levels of depolarization. The persistent interruption of PV-IN firing can potentially trigger persistent activity in postsynaptic PYRs via disinhibition.

### PV-IN neurons display type 2 excitability

Early models of CA1 fast-spiking PV-INs (Wang and Buzsáki, 1996) as well as more recent ones (Ferguson et al., 2013) exhibited Hodgkin's type 1 excitability, characterized by an *f*/*I* curve that theoretically starts at arbitrarily low frequencies (Hodgkin, 1948; Izhikevich, 2007; Knowlton et al., 2020). On the other hand, models of fast-spiking neurons in the mouse somatosensory cortex (Erisir et al., 1999), like their experimental counterparts, exhibited type 2 excitability with a minimum frequency of tens of hertz. Type 2 excitability provides a mechanism for “stuttering” that is distinct from elliptic bursting, although in the presence of noise, they appear similar. The presence of stuttering in the transition region between quiescence or transient firing and tonic firing implies type 2 excitability. In the bistable region (Fig. 7*C*2), noise can induce switches between quiescence and tonic firing (Izhikevich, 2007; Schreiber et al., 2009). Bistability based solely on type 2 excitability is not a reliable substrate for persistent interruption, as shown in Figure 7. An extension of the Erisir model (Golomb et al., 2007) examined different parameter settings regarding the contribution of the Na^+^ window current that controls whether inward or outward currents dominate at steady state in the range of membrane potentials traversed during the interspike interval. Dominant inward currents produce type 1 excitability, and dominant outward currents produce type 2 excitability (Prescott et al., 2008). It is possible that PV-INs exhibit both types of excitability depending upon their exact mix of conductances, but our data suggest predominantly type 2 excitability in the mEC (Tikidji-Hamburyan et al., 2015; Martínez et al., 2017; Via et al., 2022) and area CA1 (Figs. 1, 2).

### Generalized role of K_V_1 in persistent interruption and transient firing

K_V_1 channel blockers eliminated the delay to first spike observed at values of *I*_app_ near the threshold to evoke tonic firing in layer 2/3 mouse barrel cortex (Goldberg et al., 2008). These delays result from the inactivation of K_V_1 channels and are commonly observed in neocortical PV-INs (Erisir et al., 1999; Golomb et al., 2007) but not in the mEC (Via et al., 2022) or CA1 PV-INs (Fig. 1*B,C* and Chamberland et al., 2023), and a mixed phenotype appears to be observed in striatal PV-INs (Bracci et al., 2003). Modeling suggests that a smaller K_V_1 window current at rest eliminates the delays by making less outward current available, favoring spiking. In the (Golomb et al., 2007) model after a current step, it takes time for K_V_1 to inactivate, and therefore, there is a delay in firing. In our model, there is less K_V_1 at rest, so our model starts to fire immediately during a current step, but for sufficiently small current steps, enough inactivation is removed from K_V_1 during the AHPs to silence the neuron. Therefore, instead of delays at values of *I*_app_ near the threshold for tonic firing, in CA1 (Fig. 1*B,C*; Chamberland et al., 2023) and the mEC (Via et al., 2022), transient firing is observed in which one or more spikes are emitted before the neuron falls silent. In CA1, the model therefore predicts that K_V_1 blockers would convert the transient firing to tonic firing as in Sciamanna and Wilson (2011). Our model and the experimental observations in area CA1 broaden the repertoire of activity conferred on PV-INs by K_V_1 expression.

The mechanisms for transitions between firing and quiescence (Chamberland et al., 2023) are similar to those observed previously (Golomb et al., 2007; Sciamanna and Wilson, 2011) in models that also include the slowly inactivating K_V_1 current, but distinct from those in another model (Via et al., 2022). The Via model exhibits a less robust persistent interruption of firing due to the subcritical HB underlying type 2 excitability, which does not involve K_V_1 current whose slow time scale protects the interruption from termination by noise. Therefore, K_V_1 not only enables persistent interruption, but it also contributes to type 2 excitability and may in some cases be required, as in striatal PV-INs (Sciamanna and Wilson, 2011).

In the absence of additional information, Via et al. (2022) modeled the transient firing as terminating due to slow activation of a K^+^ current they called K_V_1, but whose characteristics more closely resemble the slowly activating K_V_7 (Prescott and Sejnowski, 2008). Experiments using K_V_1 and K_V_7 blockers are required to determine which current is responsible for cessation of firing of PV-INs in the mEC, but it seems likely that the mEC interneurons, like their cortical and hippocampal counterparts, rely on K_V_1 rather than K_V_7 to regulate firing activity near threshold. Thus, we predict that robust persistent interruption of firing can be observed in mEC and neocortical PV-INs, just as in CA1. Moreover, K_V_1 channels are expressed in many other neurons including dopaminergic neurons (Fulton et al., 2011) and deep cerebellar neurons (McKay et al., 2005). Therefore, it is possible that other neuronal types may exhibit some of the features enabled by K_V_1 such as transient firing, firing delays, elliptic bursting, and persistent interruption of firing.

### Inhibitory dynamics controlling CA1 activity in vivo

CA1-PYRs often function as place cells that fire when an animal is in a particular area of their environment (O’Keefe and Dostrovsky, 1971) and have been suggested as a model for general episodic memory (Redish, 1999). Local CA1 inhibitory circuits contribute to place cell firing (Royer et al., 2012; Grienberger et al., 2017; Zutshi et al., 2022; Valero et al., 2022), and interneurons are thought to coordinate sequences of place cells (Udakis et al., 2020; Valero et al., 2022; Zutshi et al., 2022). The influence of different subtypes of interneurons on place cell firing varies as a function of place field position. This is best exemplified by the progressive shift in the influence of PV → SST INs over CA1-PYR firing when the animal traverses a place field (Royer et al., 2012), for which PV-mediated inhibitory influence on CA1-PYR firing is highest at place field entry and gradually diminishes (Royer et al., 2012). This results in a progressive shift in perisomatic to dendritic inhibition, and it is remarkable that the CA1 circuit is intrinsically wired to support this shift (Pouille and Scanziani, 2004).

The persistent interruption of firing is an attractive cellular mechanism to explain how the dampening of PV-mediated inhibition could enhance CA1-PYR firing in their place field (Royer et al., 2012; Valero et al., 2022), likely complementing the short-term synaptic dynamics in the circuit (Pouille and Scanziani, 2004). While any source of inhibition that is sufficiently strong can theoretically interrupt PV-IN firing, recent work (Chamberland et al., 2024) identified a population of bistratified somatostatin-positive (*Sst*) INs that expresses the tachykinin precursor 1 (*Tac1*) gene. These *Sst;;Tac1*-INs preferentially synapse onto FS-INs and suffice to interrupt their firing, unlike two populations of *Sst*-expressing oriens-lacunosum–moleculare INs that predominantly target CA1-PYRs and for the most part avoided FS-INs. Thus, *Sst;;Tac1*-INs potentially control persistent interruption in PV-INs and the resultant lasting disinhibition of CA1-PYRs. The stimulus-precipitating persistent activity in this case would be the excitation of *Sst;;Tac1*-INs, known to fire after PV-INs during hippocampal CA3 activity (Chamberland et al., 2024). Increasing the depolarizing current injected into PV-INs abolished the ability of IPSPs to interrupt firing (Chamberland et al., 2023). Therefore, it is likely that only the most weakly excited PV-INs would be interrupted by *Sst;;Tac1*-INs, effectively disinhibiting subsets of CA1-PYRs. Disinhibition of CA1-PYR subsets could also be achieved through variable interruption duration across PV-INs (Börgers et al., 2010) or by nonuniformly distributed synaptic inhibition strength provided by *Sst;;Tac1*-INs to PV-INs (Chamberland et al., 2024). The simulations in Figure 7 used the standard deviation in current noise that produced a standard deviation of 5 mV in the membrane potential. This closely approximates a known estimate of the standard deviation of the membrane potential in vivo during wakefulness (2–6 mV; Destexhe and Rudolph-Lilith, 2012). Moreover, in vivo experiments reported in Figure 3 of Chamberland et al. (2023) show that optogenetic stimulation of SST neurons results in silence periods that are longer than the average ISI and persist beyond the optogenetic stimulation duration, suggesting that SST neurons might be able to induce persistent interruption in intact animals. The exact conditions under which this phenomenon might occur in vivo will need to be identified in the future, but we hypothesize that persistent interruption enabled by slow inactivation of K_V_1 could be a substrate for a memory trace, in large part due to its robustness in the presence of noise (Fig. 7).

## Synthesis

Reviewing Editor: Arvind Kumar, KTH Royal Institute of Technology Decisions are customarily a result of the Reviewing Editor and the peer reviewers coming together and discussing their recommendations until a consensus is reached. When revisions are invited, a fact-based synthesis statement explaining their decision and outlining what is needed to prepare a revision will be listed below. The following reviewer(s) agreed to reveal their identity: NONE. Note: If this manuscript was transferred from JNeurosci and a decision was made to accept the manuscript without peer review, a brief statement to this effect will instead be what is listed below.

Both reviwers are happy with the revision. But on of the reviewer is not entirely happy with some of the text in the discussion. Once that is fixed we can make the final decision on this manuscript.

Reviewer #1

I do not agree with their new paragraph in Discussion (lines 575-581). There are other parameters that might affect their in vivo model. And their model sets the noise s.d. to 165 pA while their IPSP (minor: line 773: IPSC?) is 100 pA. It is hard to know if these values are comparable to in vivo values. I also do not agree that the SST activation silencing PVs for some moderate time is proof of their conclusion.

## Author Response

Thank you for the opportunity to resubmit. We have addressed reviewer 1's concerns as follows, and we hope the manuscript is now suitable for publication in eNeuro.

Reviewer #1 1) I do not agree with their new paragraph in Discussion (lines 575-581). There are other parameters that might affect their in vivo model.

We clarified this paragraph as described in 2 and 3 below.

2) And their model sets the noise s.d. to 165 pA while their IPSP (minor: line 773: IPSC?) is 100 pA. It is hard to know if these values are comparable to in vivo values.

We apologize for our lack of clarity. We reworded the Discussion sentence relevant to the in vivo model as follows: "The simulations in Fig. 7 used the standard deviation in current noise that produced a standard deviation of 5 mV in the membrane potential. This closely approximates a known estimate of the standard deviation of the membrane potential in vivo during wakefulness (2-6 mV) (Destexhe and Rudolph-Lilith 2012b)." 3) I also do not agree that the SST activation silencing PVs for some moderate time is proof of their conclusion.

We softened the language to read as follows: "Moreover, in vivo experiments reported in Figure 3 of Chamberland et al. 2023 show that optogenetic stimulation of SST neurons results in silence periods that are longer than the average ISI and persist beyond the optogenetic stimulation duration, suggesting that SST neurons might be able to induce persistent interruption in PV-INs in animals. The exact conditions under which this phenomenon occurs in vivo will need to be identified in the future, but we hypothesize that persistent interruption enabled by slow inactivation of KV1 could be a substrate for a memory trace, in large part due to its robustness in the presence of noise (Fig. 7).
