# Supplementary figures and images for: Persistent Interruption in Parvalbumin-Positive Inhibitory Interneurons: Biophysical and Mathematical Mechanisms
Source: eNeuro. 2024 Jul 4;11(7):ENEURO.0190-24.2024. doi: 10.1523/ENEURO.0190-24.2024 (PMC11236577; doi:10.1523/ENEURO.0190-24.2024)

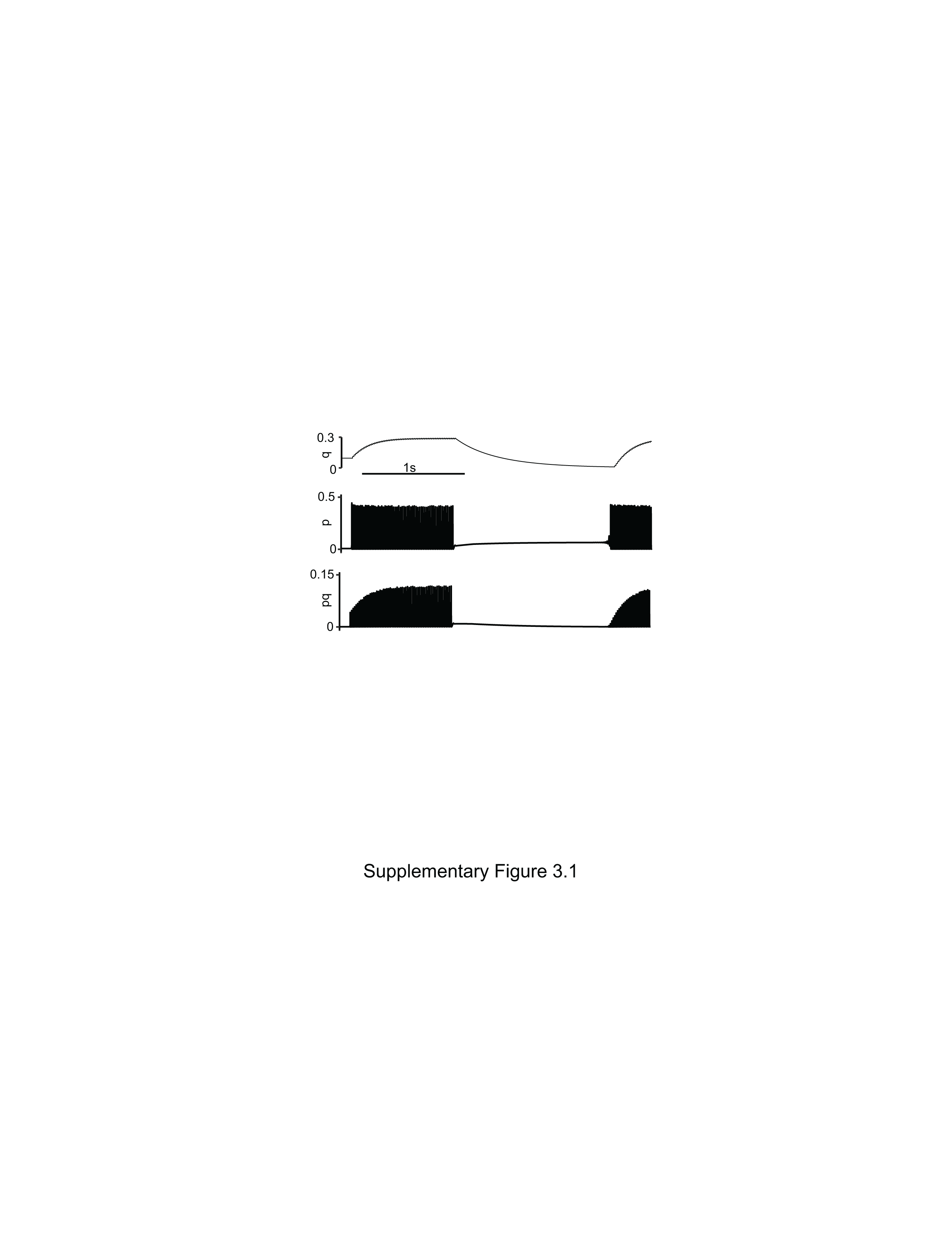

Supplement: Figure 3.1 — Fig. 3B repeated with panels added for p and pq. Download Figure 3.1, TIF file. [file eneuro-11-ENEURO.0190-24.2024-s001.tif]

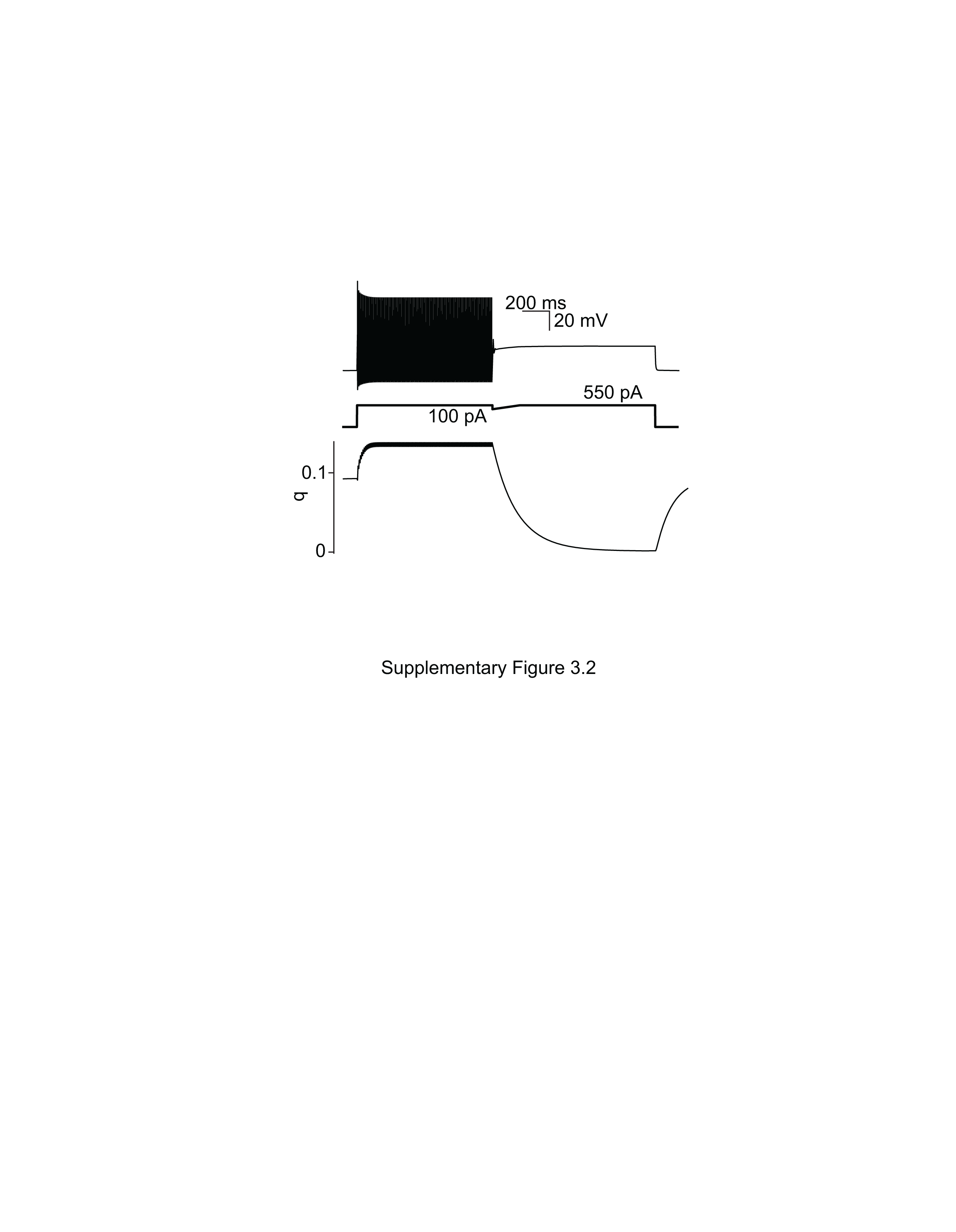

Supplement: Figure 3.2 — Fig 3AB rerun at 32o from a baseline of 23°C with Q10 of 2. Persistent interruption is robust at higher temperatures, consistent with Chamberland et al 2023. The major effect of increasing the temperature was to shift the Hopf bifurcation to more hyperpolarized potentials. Download Figure 3.2, TIF file. [file eneuro-11-ENEURO.0190-24.2024-s002.tif]
